# A Systematic Review of the Gene–Lifestyle Interactions on Metabolic Disease-Related Outcomes in Arab Populations

**DOI:** 10.3390/nu16152519

**Published:** 2024-08-01

**Authors:** Maria M. AlAnazi, Eduard Flores Ventura, Julie A. Lovegrove, Karani Santhanakrishnan Vimaleswaran

**Affiliations:** 1 Hugh Sinclair Unit of Human Nutrition, Department of Food and Nutritional Sciences, University of Reading, Reading RG6 6DZ, UK; m.m.alanazi@pgr.reading.ac.uk (M.M.A.); j.a.lovegrove@reading.ac.uk (J.A.L.); 2 Department of Human Nutrition, College of Health Sciences, Qatar University, Doha P.O. Box 2713, Qatar; 3 Institute of Agrochemistry and Food Technology—Spanish National Research Council (IATA-CSIC), 46980 Valencia, Spain; eduard.flores.ventura@hotmail.com; 4 Institute for Food, Nutrition and Health (IFNH), University of Reading, Reading RG6 6AH, UK

**Keywords:** Arab populations, gene–lifestyle interaction, single nucleotide polymorphism (SNP)

## Abstract

The increased prevalence of metabolic diseases in the Arab countries is mainly associated with genetic susceptibility, lifestyle behaviours, such as physical inactivity, and an unhealthy diet. The objective of this review was to investigate and summarise the findings of the gene–lifestyle interaction studies on metabolic diseases such as obesity and type 2 diabetes in Arab populations. Relevant articles were retrieved from a literature search on PubMed, Web of Science, and Google Scholar starting at the earliest indexing date through to January 2024. Articles that reported an interaction between gene variants and diet or physical activity were included and excluded if no interaction was investigated or if they were conducted among a non-Arab population. In total, five articles were included in this review. To date, among three out of twenty-two Arab populations, fourteen interactions have been found between the *FTO* rs9939609, *TCF7L2* rs7903146, *MC4R* rs17782313, and *MTHFR* rs1801133 polymorphisms and diet or physical activity on obesity and type 2 diabetes outcomes. The majority of the reported gene–diet/ gene–physical activity interactions (twelve) appeared only once in the review. Consequently, replication, comparisons, and generalisation of the findings are limited due to the sample size, study designs, dietary assessment tools, statistical analysis, and genetic heterogeneity of the studied sample.

## 1. Introduction

Non-communicable diseases (NCDs) have been shown to account for 73% of the global mortality rate worldwide [1]. The global prevalence of NCDs, such as obesity and type 2 diabetes (T2D), is increasing, and the study of the global burden of metabolic diseases reported that the mortality rate for obesity and T2D has risen from 2000 to 2019, with an annual increase of 0.8% and 0.5% for T2D and obesity, respectively [2]. Obesity is a progressive chronic disease that plays a significant role in the acceleration of T2D pathogenesis through the development of insulin resistance [3,4]. Metabolic diseases are multifactorial in nature, with their aetiology influenced by the interaction between genetic predisposition and environmental factors such as physical activity (PA) and diet [5,6].

In Arab populations, which, according to the 2023 census, was estimated to be around 473 million people [7,8,9], there has been a massive shift in the prevalence of NCDs [10]. Additionally, the prevalence of obesity and T2D among Arab adults ranged between 8% and 24% [11], with a threefold increase observed between 1975 and 2016 [12]. Although Arabs share a common language and history, the Arab population is considered a heterogeneous ethnic group due to significant demographic, geographic, socioeconomic, and political diversity [13,14]. Irrespective of the economic diversity that exists across these countries, the increased incidence of obesity and T2D is primarily associated with lifestyle behaviours, such as smoking, physical inactivity, and changes in dietary practices [15,16]. Genetic association studies on obesity were conducted in 12 Arab countries, which revealed a total of 76 single nucleotide polymorphisms (SNPs) in 49 genes that were found to be associated with obesity and its related traits [12]. Among these SNPs, 55 were previously reported in different populations, while 21 SNPs were distinctively associated with obesity among Arab populations only [12]. Additionally, 57 SNPs in 32 genes associated with T2D were reported in 13 Arab populations [17,18,19,20].

It should be noted that the genetic heritability of obesity and diabetes ranges between 30% and 70% [5], with approximately 700 genetic loci associated with obesity and 400 with T2D [3]. However, research on gene–diet interactions has revealed that environmental modifications can mitigate the genetic susceptibility to obesity and T2D [5]. Furthermore, lifestyle changes, such as weight management programs, have demonstrated improved glycaemic control in diabetic patients and a preventive effect on impaired glucose tolerance in individuals with prediabetes [21]. Regrettably, while gene–diet interactions have been extensively studied in Western populations, this area remains understudied in other regions of the world, particularly in the Middle East [22,23]. This systematic review comprehensively examines, evaluates, and synthesises the findings of gene–lifestyle interaction studies conducted in Arab populations.

## 2. Materials and Methods

### 2.1. Inclusion and Exclusion Criteria and Search Strategy

**Figure 1:**
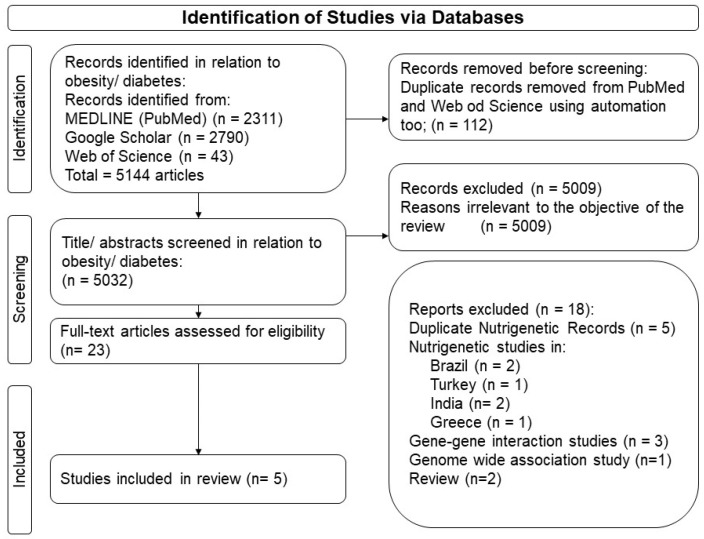
PRISMA flow chart illustrating the exclusion criteria and studies selection.

The Arab population refers to the member nations of the Arab League, which include 22 countries spread over 13 million km^2^ on the Arabian Peninsula and Northern Africa, including Algeria, Bahrain, Comoros, Djibouti, Egypt, Iraq, Jordon, Kuwait, Lebanon, Libya, Mauritania, Morocco, Oman, the Palestinian Authority, Qatar, Saudi Arabia, Somalia, Sudan, Syria, Tunisia, the United Arab Emirates, and Yemen. We included articles that investigated the interaction between SNPs and dietary factors, smoking, or physical activity on the primary traits related to metabolic diseases that were conducted in Arab populations. The studies included both observational and dietary intervention studies and those studies that were written in English. We excluded articles that did not investigate gene–lifestyle interactions in humans or were not based in any of the 22 Arab countries. To explore the published articles, a literature search was conducted on the PubMed (National Library of Medicine) database, Google Scholar, and Web of Science, starting from the earliest indexing date to the end of January 2024 (Figure 1). To ensure literature saturation; the references from relevant articles were reviewed, and researchers, M.M.A. and E.F.V., independently performed search strings. Supplementary Table S1 presents the search strings used.

### 2.2. Data Collection, Extraction, and Synthesis Methods

The studies were identified by a single investigator (M.M.A.) and verified by a second investigator (E.F.V.) through independent search strings. Titles and abstracts were screened blindly to evaluate them against the pre-established inclusion criteria. This was followed by full-text screening and discussions between M.M.A. and E.F.V. The reviewers ensured consistency in the data extraction process, which encompassed the publication year, participant location or ethnicity, sample size, study design, exposure, gene name, reference SNP ID, genotype, and minor allele. A narrative synthesis approach was employed to compile data focusing on populations; factors related to physical activity and diet; study designs; SNPs; obesity- and diabetes-related traits; and *p*-values for gene–lifestyle interactions concerning the primary outcomes associated with obesity [e.g., body mass index (BMI), adiposity, and waist-to-hip ratio (WHR)] and diabetes traits such as glycated haemoglobin (HbA1c), fasting glucose, or insulin secretion. *p*-values for interactions were utilised to evaluate the significance level of gene–diet and gene–physical activity interactions in relation to obesity and diabetes traits. To synthesise the studies, they were sub-grouped based on the studied exposures, further categorised by traits of obesity or diabetes, and ultimately grouped based on SNPs.

**Figure 2:**
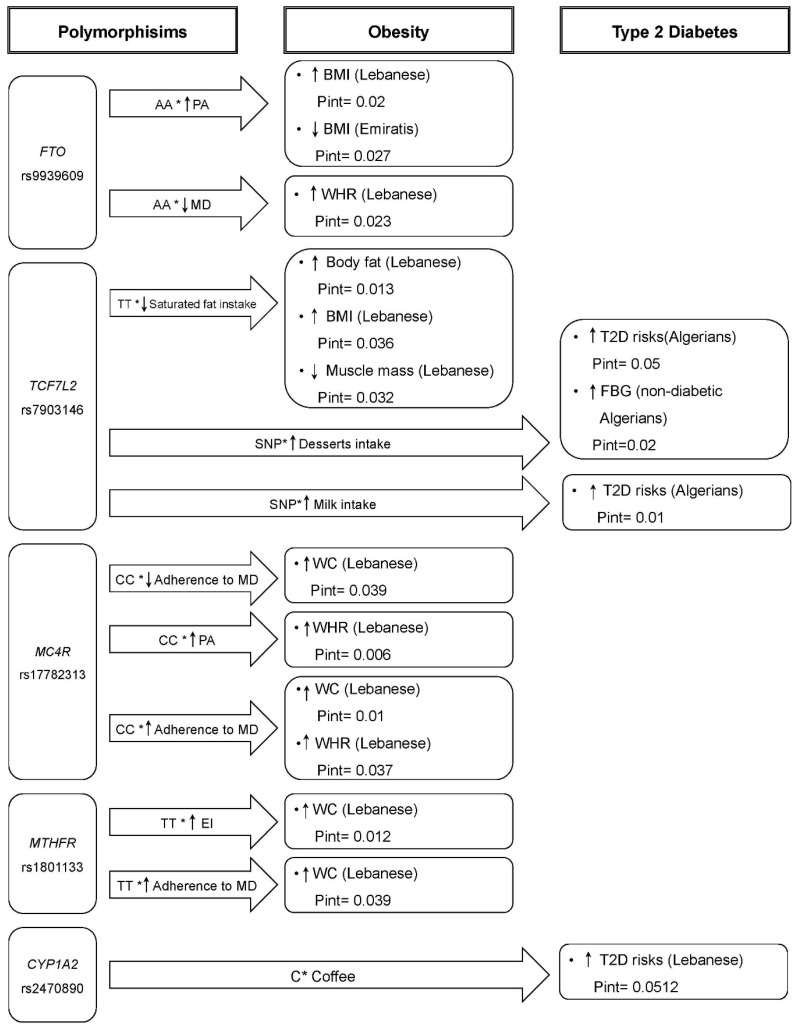
Interactions of polymorphisms with diet and physical activity on obesity and diabetes traits. ↑ indicates an increase, ↓ indicates a decrease, * indicates an interaction.

All identified gene–diet and gene–physical activity interaction *p*-values were tabulated with a summary of the study design, ethnicity, sample size, gene name, SNP (rsID), and assessment tool (Table 1), whereas only significant interactions with the exposure and outcome variables were presented in (Figure 2).

### 2.3. Quality and Risk of Bias Assessments

The present article follows the Synthesis Without Meta-analysis (SWiM) in Systematic Reviews: Reporting Guideline recommendations [29]. The risk of bias (RoB) was evaluated using the appraisal tool for Cross-Sectional Studies (AXIS) [30], the RoB in Non-randomized Studies—of Interventions (ROBINS-I) assessment tool [31], and the Cochrane risk of bias tool for randomized trials (RoB 2) [32]. The outputs for the RoB can be found in Supplementary Tables S2–S4. This systematic review was registered at the International Prospective Register of Systematic Reviews (PROSPERO) (ID: CRD42023405449).

## 3. Results and Discussion

### 3.1. Outputs from the Search Strategy

The search strategy produced a total of 5144 results. A total of 112 duplicate articles from PubMed and Web of Science were removed using automated tool “Rayyan”. After reviewing the titles and abstracts, 5009 articles were excluded, resulting in 23 articles, of which 18 were excluded after reading the full text (Figure 1). Only five articles were deemed suitable for inclusion in this review, of which three studies were conducted on the Lebanese population [25,26,28]. The remaining two studies were conducted on Algerian [27] and Emirati [24] populations, respectively.

### 3.2. Gene–Lifestyle Interactions on Obesity and Diabetes Traits

#### 3.2.1. Gene–Diet and Gene–Physical Activity Interactions on Obesity Traits

##### Interaction between SNP *TCF7L2* rs7903146 and Diet on Obesity in the Lebanese Population

Transcription Factor 7 Like 2 (*TCF7L*) SNP rs7903146 is one of the most extensively studied SNPs in relation to obesity and T2D. The association of this SNP with the risk of T2D has been previously established in various populations [33,34,35], and evidence suggests that it may interact with diet to impact obesity indicators [34,36]. 

In a cross-sectional study involving 308 Lebanese adult participants (age > 18 years), an interaction between SNP rs7903146 and diet was observed on obesity-related outcomes [25]. The study reported significant interactions between the intake of energy from saturated fat and the risk allele “T” on higher body fat (P_interaction_ = 0.013), BMI (P_interaction_ = 0.016), and lower muscle mass (P_interaction_ = 0.032) (Table 1 and Figure 2). However, TT homozygous individuals showed a higher risk of obesity, even when their saturated fat consumption was lower. No interaction of the SNP with total dietary fat, monounsaturated fatty acid (MUFA), or polyunsaturated fatty acid (PUFA) intake was observed. 

The mean daily energy intake in this study involving Lebanese adults was 3600 ± 2029 kcal/day, with fat intake contributing to 39% of the daily total energy intake [37]. It is recommended that saturated fat intake should not exceed 10% of daily energy [38], which is according to Recommended Daily Allowances (RDAs) ranges between 1900 and 2220 kcal/day for women and 2300 and 2900 kcal/day for men [38]. Additionally the Lebanon food-based dietary guidelines have set the daily caloric intake as 2000 kcal/day [39]; however, in this study on Lebanese adults, the saturated fat intake ranged from <36 g (16%) to >44 g (20%), which is noticeably higher than the recommendations from the published guidelines (<10%) [40]. Besides highlighting the role of genetic heterogeneity, the study also demonstrated the significance of reducing the consumption of saturated fat intake in the Lebanese population irrespective of genetic susceptibility. Several studies have examined the interaction of the SNP rs7903146 with dietary fat intake on obesity traits; however, the findings have been inconsistent. Among 771 obese Europeans, an intervention study found that participants who were TT homozygous for SNP rs7903146 and had a high-fat diet experienced lower weight loss compared to TT carriers on a low-fat diet [41]. Another interventional study involving 588 overweight and obese Americans reported that there was a significant interaction between SNP rs7903146 and dietary fat on lean mass [42]. The decrease in lean mass was significant only in common homozygous individuals (CC) on a low-fat diet (20–25% of daily energy) compared to the high-fat diet group (40–45% of daily energy). However, among those with the rare homozygous TT genotype, there was no significant difference in lean mass loss between the two diet groups [43]. In a study of 309 German adults at risk of T2D, a lifestyle intervention consisting of a low-fat (<30%), low-saturated fat (<10%), and high-fibre diet showed that SNP rs7903146 risk allele “T” carriers had reduced BMI values compared to other genotypes [43].

The results indicated that SNP rs7903146 risk allele “T” carriers who were on high-fat or high-saturated fat diets may experience increased obesity risks in Lebanese (n = 308) and European populations (n = 771) [25,41]; yet, this evidence was not identified when the same interaction was studied among Americans (n = 588) [43]. Evidence from the German population (n = 309) reported an improved effect on lowering obesity risks when TT carriers were put on a low-fat, low-saturated fat, and high-fibre diet [43], therefore indicating the need for similar interventions among Lebanese populations.

##### Interaction between *MC4R* SNP rs17782313 and Diet or Physical Activity on Obesity in the Lebanese Population

The Melanocortin 4 Receptor (*MC4R*) gene is responsible for regulating food intake and energy balance [44], and the *MC4R* gene SNP rs17782313 has been reported to be associated with a higher risk of obesity and insulin resistance [45,46].

A cross-sectional study investigated the interaction between multiple SNPs and the Mediterranean diet in 392 healthy adult Lebanese participants (age 34.7 ± 11.5 years) in relation to obesity outcomes [26]. The study found an interaction between the risk allele “C” of the SNP rs17782313 and a weak adherence to the Mediterranean diet (MD) with a higher waist circumference (WC) (P_interaction_ = 0.039). However, “C” allele carriers with strong adherence to the MD also exhibited high WC values (P_interaction_ = 0.01). Similarly, CC homozygotes with a strong adherence to the MD showed a greater waist-to-hip ratio (WHR) (P_interaction_ = 0.037) (Table 1 and Figure 2) [26]. Although an interaction was observed between the SNP rs17782313 and physical activity for the WHR (P_interaction_ = 0.006) (Table 1 and Figure 2), high levels of physical activity did not attenuate the association between the risk allele “C” with a higher WHR. These results suggest that carrying two copies of the risk allele “C” is associated with obesity among Lebanese participants regardless of their physical activity level and MedDiet score. It is worth noting that the study only included healthy individuals with a mean BMI of 25.15 ± 4.66 kg/m^2^ (normal and overweight only) [26], which might have introduced selection bias due to the exclusion of obese participants. In addition, although the researchers employed a validated method to assess physical activity [short version of the International Physical Activity Questionnaire (IPAQ)] [26], only the validity and reliability of the Arabic long version of IPAQ have been previously assessed among Arab populations [47], while the language used in this study was not specified [26]. Therefore, it can be assumed that a translated version of IPAQ was utilised, which has not been validated in this population [48]. Furthermore, a systematic review on the validity of the short version of the English IPAQ revealed an 84% overestimation of physical activity, suggesting weak evidence supporting the use of the short version of IPAQ as an indicator of physical activity [49]. In this study, 48% of the participants were classified as moderately active, 30% as highly active, and only 22% with a low activity level [26], whereas a previous study that used self-reported exercise type and frequency to assess leisure time physical activity using the updated Compendium of Physical Activities reported that 67% of the participants were inactive [50]. These findings support evidence of the overestimation of the physical activity level when using the short version of the IPAQ.

In contrast, a systematic review of fourteen studies on individuals from different non-Arabic countries (Indian, German, Canadian, Swedish, Chinese, Finnish, Spanish, Danish, and Czech) noted that adherence to the MD can influence the association between the *MC4R* SNP rs17782313 and the risk of developing T2D and obesity, where increased adherence to the MD was associated with lower risks of obesity and T2D [51]. However, studies investigating the interaction between SNP rs17782313 and diet on cardiometabolic traits specifically in Middle Eastern populations have only been conducted in Iran. A study involving 188 men and women from Iran reported that individuals homozygous for the risk allele (CC) exhibited an interaction with low adherence to healthy eating patterns, such as the Healthy Eating Index-2015 (HEI) and the Diet Quality Index-International (DQI-I), resulting in higher fasting blood glucose (FBG) levels and systolic and diastolic blood pressures in obese Iranian men [52]. Another interaction was observed between the CC genotype and moderate adherence to the HEI, leading to higher levels of LDL-C among obese Iranian women [52]. In a study involving 288 obese Iranian participants, CC carriers with high adherence to the Dietary Approach to Stop Hypertension (DASH) diet showed lower serum glucose concentrations among men. Additionally, low adherence to the MD was associated with lower agouti-related peptide (AgRP) concentrations in men with the CC genotype [53]; AgRP is an antagonist, and its overexpression has been linked to obesity [54]. Notably, the MD did not show an association with *MC4R* rs17782313 [53]. In a study of overweight and obese Iranian women (n = 266), an interaction between the C allele and high adherence to the DASH diet was found to lower the cardiovascular disease risk [55]. Additionally, a study in 3850 adult Iranians observed that increased adherence to healthy dietary patterns showed an interaction with the “C” allele on lower obesity risk in this population [56]. These findings cannot be compared with the results from the Lebanese study [26], given that the Lebanese study did not include obese participants and did not test for the gene–MD adherence interaction on obesity in both sexes.

##### Interaction between *MTHFR* SNP rs1801133 and Diet on Obesity in the Lebanese Population

In a cross-sectional study of 392 healthy adult Lebanese individuals, an interaction was observed between the SNP rs1801133 and energy intake (EI) on WC (P_interaction_ = 0.012) [26]. Individuals carrying two copies of the risk allele “T” exhibited higher WC even when consuming a low daily energy diet (≤1831 kcal), with the WC measuring 86.7 ± 15.2 cm for TT carriers compared to 86 ± 13 cm for CC carriers. Although statistically significant, these differences have little clinical importance. A similar finding was observed for the WC when examining the impact of increased adherence to a MD (P_interaction_ = 0.039), where the adherence to a MD did not protect against obesity for TT carriers in this population (Table 1 and Figure 2). These findings suggest that the risk allele of SNP rs1801133 was associated with obesity risk among Lebanese individuals regardless of their consumption of a low-energy diet. The methylenetetrahydrofolate reductase (*MTHFR*) gene encodes methylenetetrahydrofolate reductase, which plays a crucial role in folic acid metabolism and contributes to DNA methylation [57]. Methylenetetrahydrofolate reductase aids in the conversion of homocysteine to methionine and, subsequently, into S-adenosylmethionine. Studies have indicated that carrying the risk allele “T” of *MTHFR* SNP rs1801133 leads to impairment of folic acid metabolism, resulting in higher plasma homocysteine levels and lower serum folate levels [57,58]. A recent meta-analysis of fourteen studies conducted on North Americans, Europeans, and Asians (710 cases/607 controls) reported a significant association between increased homocysteine levels and obesity regardless of dietary habits, nutritional status, insulin resistance, medical conditions, or genetic background [59]. These studies may partly explain the inability to protect against obesity among Lebanese individuals carrying the risk allele “T” of SNP rs1801133, despite adhering to a healthy diet [57,58,59].

##### Interaction between *FTO* rs9939609 and rs1558902 and Diet or Physical Activity on Obesity in the Lebanese and Emirati Populations

The Fat Mass and Obesity Associated (*FTO*) gene, which is profoundly expressed in the hypothalamus, has been shown to be essential in the encoding for the protein involved in the oxidative reactions, fatty acid metabolism, and energy metabolism [57,60,61,62]. *FTO* genetic variants such as rs9939609, rs1121980, and rs1558902 have been shown to be associated with obesity and its related outcomes [63,64].

In a cross-sectional study of 392 healthy adult Lebanese individuals, interactions were observed between *FTO* SNP rs9939609 and physical activity levels on higher BMI (P_interaction_ = 0.02) (Table 1 and Figure 2). Despite having high levels of physical activity, people with the AA genotype had the highest BMI (25.6 ± 4.3 kg/m^2^) [26]. Additionally, an interaction was found between the AA genotype of SNP rs9939609 and low adherence to the MD on increased WHR (P_interaction_ = 0.023 for low MD scale) (Table 1). However, the validity of the interaction between SNP rs9939609 and high PA on BMI is questionable, since the study excluded obese individuals, and the interactions were not consistent across all obesity indicators such as the BMI, WC, and WHR [26]. Furthermore, using BMI as the sole indicator of obesity may not be accurate, especially when no sex- or ethnicity-specific cutoffs have been established [65]. Moreover, the interaction of SNP rs9939609 with lifestyle factors on obesity outcomes was only adjusted for the following confounders: age, sex, and physical activity. However, it should be noted that 30% of participants were cigarette smokers, and 65% consumed alcohol. Additionally, the prevalence of waterpipe smoking (hookah) was found to be 37% among Lebanese individuals, making it the highest in the Middle East [66]. Considering that hookah smoking has also been strongly associated with obesity [67], future gene–diet interaction studies should also consider adjusting for smoking. Another factor associated with obesity in the Eastern Mediterranean region is income, which has also been linked to low adherence to the MD [68,69]. Previous studies emphasised the importance of controlling or adjusting for all potential covariates when determining the relationship between an exposure and outcomes [70,71]. Overlooking these potential covariates may have influenced the results of the gene–diet/gene–physical activity interactions in the present study [26]. 

In contrast, a cross-sectional study of 308 adult Lebanese individuals (both obese and non-obese) found no significant interactions between dietary factors, including carbohydrates, fat, protein, saturated fat, MUFA and PUFA, and *FTO* SNPs rs9939609 or rs1558902 on the BMI, body fat, or muscle mass [25]. This lack of interaction between SNP rs9939609 and dietary intake is consistent with the aforementioned study of 392 Lebanese participants, where the daily energy intake and percentages of fat, carbohydrate, and protein intake did not show any interactions with the BMI, WHR, and WC [26]. Similarly, a study on Polish women (n = 201) also reported no interaction between SNP rs9939609 and PA on the BMI levels [72]. However, in an adult Turkish population (n = 400), rs9939609 risk allele “A” showed an interaction with low PA on obesity risk [73]. It should be noted that the absence of interaction between *FTO* SNPs and lifestyle factors in the Lebanese population may be attributed to the relatively small sample size (n = 308), which is a common challenge in candidate gene studies [74].

Although both studies of Lebanese populations were cross sectional-studies [25,26] and utilised a food frequency questionnaire (FFQ), one study employed a 157-item [26], while the other an 80-item, FFQ [25]. Research has indicated that FFQs with a larger number of items yield more precise results [75,76], potentially influencing the reliability of reported interactions among Lebanese participants [25,26]. Furthermore, it is essential to consider the historical context of Lebanon when discussing genetics, as the Lebanese population is multi-ethnic [77]. Immigration trends throughout history have resulted in a highly heterogeneous population in Lebanon [78,79]. For example, the last racial census conducted in 1932 categorised the population into Maronites, Druzes, Greek Orthodox, Greek Catholics, Sunnites, Shiites, Armenians, and other minority groups [77,80]. Therefore, the genetic heterogeneity and lack of available data on ethnicity distribution may have contributed to the population stratification in the studied samples, which is a common pitfall in candidate gene studies [81]. 

A case–control study was conducted among 414 obese and non-obese adult Emirati participants (mean age = 55.7 ± 13.14 years) to explore the interaction of *FTO* and *VDR* SNPs with physical activity on obesity outcomes [24]. The study reported an interaction between the “A” risk allele of SNP rs9939609 and high physical activity levels with lower BMI levels (P_interaction_= 0.027) after adjusting for age and systolic blood pressure (Table 1 and Figure 2) [24]. These findings suggest that the association between SNP rs9939609 and obesity can be modulated by physical activity levels among Emiratis. Similar observations have been demonstrated in previous studies, indicating that the genetic risk of obesity can be modulated by the levels of physical activity [25,82,83,84]. In a Spanish population, there was a significant interaction between the A allele of *FTO* SNP rs9939609 and low physical activity levels on increased BMI values (P_interaction_ = 0.001) [85]. In addition, a meta-analysis that included data from five European cohorts from Denmark, Germany, the United Kingdom, Italy, and the Netherlands reported an interaction between *FTO* SNP rs9939609 and the energy from fat, carbohydrates, and protein on obesity traits such as the BMI and WC, respectively [86]. In a cross-sectional study of 4839 Swedish adults, an interaction was observed between the SNP rs9939609 and fat intake on the BMI where TT carriers who consumed a high-fat diet and had low physical activity levels had higher BMI levels [87].

In general, *FTO* SNP rs9939609 has consistently shown a significant association with the risk of obesity in six different Arab populations (Egyptian, Saudi Arabian, Tunisian, Iraqi, Kuwaiti, and Emirati) [12]. Studies have suggested that this association may be attributed to increased energy intake, food responsiveness, and decreased satiety [88]. Therefore, it is crucial to comprehend the underlying mechanism involved in the interaction between food intake and *FTO* SNPs on obesity traits.

##### Interaction between *VDR* rs1544410 (BsmI) and Physical Activity on Obesity in the Emirati Population

The Vitamin D Receptor (*VDR*) gene encodes for the vitamin D receptor, which is a nuclear receptor abundantly expressed in various tissues and aids in vitamin D formation by binding to the active hormonal form of vitamin D (1,25-dihydroxyvitamin D) [89]. Vitamin D is involved in different biological process in the body, including skeletal metabolism, the immune system, and cell functions [90,91]. Of the several *VDR* gene polymorphisms [92], BsmI (rs1544410) has been reported to be associated with obesity and T2D in different ethnicities, including Arabs [91]. The case–control study of 414 adult Emirati participants investigated the interaction between *VDR* SNP rs1544410 and physical activity on obesity; however, no significant interaction was observed (Table 1). The study also failed to demonstrate an association between the SNP and BMI [24]. This lack of association between SNP rs1544410 and obesity traits aligns with another study that examined the association between BsmI and components of metabolic syndrome (Mets) in adult Emiratis (n = 198) [92]. The significant association between the BsmI SNP risk allele “C” and obesity-related traits was also explored in different ethnic groups, including four Arab populations, as well as Brazilian, Polish, French, Swedish, and Vietnamese populations, where the association remained consistent across all aforementioned nine cohorts [91]. It is worth noting that approximately 63% of the overweight and obese participants were diabetic; however, the results were not adjusted for diabetes status when reporting the interaction between the SNP and physical activity on the BMI [24]. Failure to adjust for appropriate confounders can result in a confounding bias, thereby negatively impacting the validity of epidemiological studies [93]. All clinical and biochemical assessments, including weight/height and lifestyle questionnaire, were not explicitly explained in the methodology, and there was no evidence that measurements were conducted by trained staff [24]. The use of self-reported height and weight can often result in an underestimation of the BMI, as participants tend to report higher height and lower weight values [94]. 

#### 3.2.2. Gene–Diet Interactions on Diabetes Traits

##### Interaction between *TCF7L2* SNPs rs7903146 and Diet on T2D in the Algerian Population

A cross-sectional study was conducted on 737 Algerian adults, including 76 individuals with diabetes (mean age = 52 ± 9.5 years) and 644 non-diabetic individuals (mean age = 43 ± 9.6 years), to investigate the interaction between the *TCF7L2* SNP rs7903146 and dietary intake on T2D outcomes [27]. Significant interactions were observed in diabetic subjects between the “T” risk allele and a high intake of desserts (consuming ≥ one per day of pastries, custards, pudding, sorbet, etc.) and high milk intake (≥twice per day) on the increased risk of T2D (P_interaction_ = 0.05 and P_interaction_ = 0.01, respectively). In non-diabetic subjects, the risk allele “T” showed an interaction with a high intake of desserts on increased FBG levels (P_interaction_ = 0.02) (Table 1 and Figure 2). The results indicate that Algerians carrying the risk allele who consume high amounts of sugar and milk are at a higher risk of developing T2D. Dietary intake in this study was assessed using a weekly FFQ consisting of 17 items, which may underestimate the actual variations in food intake [95]. However, it was not specified whether this FFQ was culturally sensitive and accurately captured the dietary intake of Algerians to enhance its validity [96]. Additionally, the researchers grouped food items to test the interactions, overlooking the nutritional profiles of these foods (macronutrients and micronutrients content), for example, pudding and custard were classified as “desserts” but they often contain “milk”, which was another category in the study [27]. In the study, the milk group did not eventually mean protein, nor did the dessert group mean refined carbohydrates and added sugars; therefore, the study findings cannot be generalised to all food items within the same food group (dairy and alternatives or processed high-fat, salt, and sugar groups) [97]. Furthermore, the quantification of portion sizes was inadequate, as the intake frequency was categorised into two groups using the median as the cutoff value (non-/low intake < median value and moderate/high intake ≥ median value) [27]. It is also important to note that the number of diabetic patients in this study was considerably low (n = 76) [27], which brings an imbalance to the number of cases and controls. The small sample size is considered a major limitation in gene–diet interaction studies [98], and hence, taking these factors into consideration, there is insufficient evidence to conclude that high milk and high dessert intake increase the risk of diabetes in Algerians carrying the risk allele [27].

##### The Interaction between *CYP1A2* SNPs rs2470890, rs2069526, rs762551, rs2472304, and rs4646427 and Diet on T2D in the Lebanese Population

The Cytochrome P450 Family 1 Subfamily A Member 2 (*CYP1A2*) gene, which belongs to the widely distributed superfamily of enzymes known as cytochrome P450, is highly expressed in the liver and plays a crucial physiological role in the metabolism of numerous endogenous substances such as sterols, testosterones, cholesterol, and bile acids, as well as the detoxification of xenobiotics like caffeine [99,100,101,102]. 

In a study including 7607 Lebanese adults (mean age = 61.3 ± 11.4 years), no significant interactions were observed between *CYP1A2* SNPs rs2069526, rs762551, rs2472304, and rs4646427 and coffee consumption in relation to T2D [28]. However, a borderline interaction was reported between the C allele of the SNP rs2470890 and coffee consumption on T2D (P_interaction_ = 0.0512) (Table 1 and Figure 2). This study identified associations between SNPs rs762551 and rs2472304 and T2D, while SNP rs2470890 was found to be associated with T2D only through its interaction with coffee intake (≥3 cups a day) [28]. This interaction could be due to the effect of SNP rs2470890 on slowing the metabolism of caffeine, resulting in its accumulation in the body, which might have reduced the death rate of beta cells and, consequently, reduced the risk of T2D [28]. No similar investigation of the interaction between *CYP1A2* SNPs and coffee or caffeine intake in relation to T2D risk has been performed in other Arab populations, highlighting the need for further studies in this area. The interaction between genes and caffeine intake has been examined in relation to obesity and chronic diseases in other populations [103]; however, the findings have been inconsistent. 

Out of the five *CYP1A2* SNPs tested in the Lebanese study [28], only SNP rs762551 was examined for its interaction with caffeine on T2D in other populations. In a hypertensive adult Italian population (n = 1180), the “C” allele of the SNP rs762551 did not show any interaction with caffeine intake in relation to the glycaemic index [104]. Conversely, a British study of non-habitual coffee drinkers (n = 30) found that the “C” allele showed an interaction with caffeine, leading to a lower postprandial glycaemic index in healthy adults [105]. However, a randomised, single-blinded trial of healthy American men (n = 20) reported that the “C” allele of the SNP rs762551 showed an interaction with caffeine and carbohydrate meals on increased postprandial glucose compared to only carbohydrate meals [106]. These results indicate that there is not sufficient evidence to determine the effect of caffeine/coffee and SNP rs762551 interaction on T2D outcomes.

### 3.3. Risk of Bias Assessment

#### Quality and Risk of Bias Assessments

According to the AXIS assessment, the included cross-sectional studies had a moderate risk of bias overall, with samples that were not representative of the studied populations [25,26,27,28]. In the study by Platt et al. [28], coffee consumption was used as a categorical variable, reducing the statistical power of the study. Furthermore, the study did not provide methodological information about the questionnaire’s development, validity, or administration method (self-administered or interview with a trained personnel). It also failed to discuss the internal limitations. The only case–control study conducted by Khan et al. among 201 obese, 115 overweight, and 98 normal weight Emirati people [24] was evaluated using the ROBINS-I tool, which revealed concerns about the potential for confounding bias in the measurement of the effect of exposure. The study examined the physical activity levels dichotomously without providing further details or a prior definition of physical activity. Additionally, when the outcome was T2D, the multiple linear regression only considered age and systolic blood pressure, failing to account for other important confounders such as sex or the BMI [24]. It is important to note that all gene–lifestyle interaction studies in Arab populations used a single SNP approach and did not calculate weighted or unweighted genetic risk scores (GRSs). GRSs can be either obtained from the external independent weight or internal marginal weight and are considered more effective approaches [107]. 

Our study had several strengths, including being the first systematic review to explore the interaction between environmental factors and different SNPs on metabolic disease-related outcomes in Arab populations. Another strength is the comprehensive search strategy aimed at including all available gene–lifestyle interactions in Arab populations. In addition, the assessed risks of bias of selected studies were conducted using standardised tools, such as AXIS and ROBINS-I, which enhanced the validity of the assessment. Regarding the limitations of the systematic review (meta-biases), the high heterogeneity across the studies precluded effective comparisons. The narrative synthesis was limited to the available evidence, and the reported results used *p*-values for interactions, which alone does not reveal the size of the effects of the interactions.

## 4. Conclusions

In summary, these findings underscore the limited number of gene–diet or gene–physical activity interaction studies conducted among Arab populations compared to other ethnic groups. This systematic review reveals that only three out of twenty-two Arab populations have been examined. Notably, the studies were predominantly conducted in Lebanon (n = 3), with one study in Algeria and another in the United Arab Emirates. Consequently, most SNPs appeared only once in this review. The heterogeneity in sample sizes, study designs, dietary assessment tools, and genetic backgrounds poses challenges for the replication, comparison, and generalisation of the findings. Given the high prevalence of metabolic diseases in Arab populations, there is a critical need for further research on gene–lifestyle interactions. Such research can provide valuable insights for developing interventions that mitigate the occurrence and impact of chronic diseases. Additionally, combining the results of nutrigenetic studies with advancements in nutrigenomics, epigenetics, metabolomics, and gut microbiome research using machine learning and artificial intelligence can facilitate the development of predictive models. These models can assist in tailoring personalised guidelines to effectively manage disease prevalence and incidence in the Arab populations.

## Figures and Tables

**Table 1:** Studies reporting significant interaction between gene variants and dietary intake or physical activity on obesity and type 2 diabetes traits among Arab populations.

Gene Symbol and Name	SNP and Nucleotide Change	Minor Allele	Study Design	Ethnicity/Sample Size	BMI Status	Dietary Intake/Physical Activity Assessment Tool	Outcome Measure	P_interaction_	References
*FTO* Fat Mass and Obesity-Associated gene	rs9939609 A/T	A	Case-control study	Emiratis 414	Normal/ overweight/obese	Lifestyle questionnaire	BMI	0.027 SNP * PA	[24]
*FTO*	rs9939609 T/A	A	Cross-sectional study	Lebanese 308	Normal/overweight/obese	Culture-specific, 80-item semi-quantitative FFQ	Body fat/BMI/Muscle Mass	-	[25]
*FTO*	rs9939609 T/A	A	Cross-sectional study	Lebanese 392	Normal/overweight	IPAQ * and 157-item, semi-quantitative FFQ	BMI	0.02 SNP * PA	[26]
							WHR	0.023 SNP * MD	
*FTO*	rs1558902 T/A	A	Cross-sectional study	Lebanese 308	Normal/overweight/obese	Culture-specific, 80-item semi-quantitative FFQ	Body fat/BMI/Muscle Mass	-	[25]
*TCF7L2* Transcription Factor 7 Like 2 gene	rs7903146 C/T	T	Cross-sectional study	Lebanese 308	Normal/overweight/obese	Culture-specific, 80-item semi-quantitative FFQ	Body fat	0.013 SNP * dietary fat	[25]
							BMI	0.016 SNP * dietary fat	
							Muscle mass	0.032 SNP * dietary fat	
*TCF7L2*	rs7903146 C/T	T	Cross-sectional study	Algerian 737	Normal/overweight/obese	Weekly FFQ	T2D risks	0.05 SNP * dessert	[27]
							T2D risks	0.01 SNP * milk intake	
							FBG	0.02 SNP * desserts	
*MC4R* Melanocortin-4 receptor gene	rs17782313 T/C	C	Cross-sectional study	Lebanese 392	Normal/overweight	IPAQ and 157-item, semi-quantitative FFQ	WC	0.039 SNP * MD	[26]
							WHR	0.037 SNP * MD	
							WHR	0.006 SNP * PA	
*MTHFR* Methylenetetrahydrofolate reductase gene	rs1801133 C/T	T	Cross-sectional study	Lebanese 392	Normal/overweight	IPAQ and 157-item, semi-quantitative FFQ	WC	0.012 SNP * dietary energy	[26]
							WC	0.039 SNP * MD	
*CYP1A2* Cytochrome P450 proteins gene	rs2470890 T/C	C	Cross-sectional study	Lebanese 7607	Normal/overweight/obese	Self-reported caffeine consumption	T2D	0.0512 SNP * Caffeine intake	[28]
	rs2069526 T/G	G							
	rs762551 A/C	C							
	rs2572304 A/G	G							
	rs4646427 C/T	C							
*VDR* Vitamin D Receptor gene	rs1544410 C/T	C	Case- control study	Emiratis 414	Normal/overweight/obese	Lifestyle questionnaire	BMI	-	[24]

* SNP: single nucleotide polymorphism; MD: Mediterranean diet; FFQ: Food Frequency Questionnaire; IPAQ: International Physical Activity Questionnaire; BMI: body mass index; T2D: type 2 diabetes; FBG: fasting blood glucose; WC: waist circumference; WHR: waist-to-hip ratio.

## Data Availability

All data has been made available in Table 1, Figure 1 and Figure 2, and in the Supplementary Materials.

## Supplementary Materials

The following supporting information can be downloaded at https://www.mdpi.com/article/10.3390/nu16152519/s1: Table S1. Search strings; Table S2. Summary outcome of the assessment with AXIS; Table S3. Assessment with AXIS; Table S4. Assessment using the risk of bias in non-randomized studies—of interventions (ROBINS-I).

## References

[1] Roth G.A., Abate D., Abate K.H., Abay S.M., Abbafati C., Abbasi N., Abbastabar H., Abd-Allah F., Abdela J., Abdelalim A. (2018). Global, regional, and national age-sex-specific mortality for 282 causes of death in 195 countries and territories, 1980–2017: A systematic analysis for the Global Burden of Disease Study 2017. Lancet.

[2] Chew N.W., Ng C.H., Tan D.J.H., Kong G., Lin C., Chin Y.H., Lim W.H., Huang D.Q., Quek J., Fu C.E. (2023). The global burden of metabolic disease: Data from 2000 to 2019. Cell Metab..

[3] Pillon N.J., Loos R.J., Marshall S.M., Zierath J.R. (2021). Metabolic consequences of obesity and type 2 diabetes: Balancing genes and environment for personalized care. Cell.

[4] Wondmkun Y.T. (2020). Obesity, insulin resistance, and type 2 diabetes: Associations and therapeutic implications. Diabetes Metab. Syndr. Obes..

[5] Vimaleswaran K.S., Loos R.J. (2010). Progress in the genetics of common obesity and type 2 diabetes. Expert Rev. Mol. Med..

[6] Temelkova-Kurktschiev T., Stefanov T. (2012). Lifestyle and genetics in obesity and type 2 diabetes. Exp. Clin. Endocrinol. Diabetes.

[7] Hamamy H.A., Al-Allawi N.A. (2013). Epidemiological profile of common haemoglobinopathies in Arab countries. J. Community Genet..

[8] Lewis B. (2002). Arabs in History.

[9] (2002). Population, Total-Arab World.

[10] Mokdad A.H., Jaber S., Aziz M.I.A., AlBuhairan F., AlGhaithi A., AlHamad N.M., Al-Hooti S.N., Al-Jasari A., AlMazroa M.A., AlQasmi A.M. (2014). The state of health in the Arab world, 1990–2010: An analysis of the burden of diseases, injuries, and risk factors. Lancet.

[11] Alzaman N., Ali A. (2016). Obesity and diabetes mellitus in the Arab world. J. Taibah Univ. Med. Sci..

[12] Younes S., Ibrahim A., Al-Jurf R., Zayed H. (2021). Genetic polymorphisms associated with obesity in the Arab world: A systematic review. Int. J. Obes..

[13] Teebi A.S. (2005). Introduction: Genetic diversity among arabs. Genet. Disord. Among Arab Popul..

[14] Mirkin B. (2010). Population Levels, Trends and Policies in the Arab Region: Challenges and Opportunities.

[15] Rahim H.F.A., Sibai A., Khader Y., Hwalla N., Fadhil I., Alsiyabi H., Mataria A., Mendis S., Mokdad A.H., Husseini A. (2014). Non-communicable diseases in the Arab world. Lancet.

[16] Sharara E., Akik C., Ghattas H., Makhlouf Obermeyer C. (2018). Physical inactivity, gender and culture in Arab countries: A systematic assessment of the literature. BMC Public Health.

[17] Abuhendi N., Qush A., Naji F., Abunada H., Al Buainain R., Shi Z., Zayed H. (2019). Genetic polymorphisms associated with type 2 diabetes in the Arab world: A systematic review and meta-analysis. Diabetes Res. Clin. Pract..

[18] Abuyassin B., Laher I. (2015). Obesity-linked diabetes in the Arab world: A review. East Mediterr. Health J..

[19] Osman W., Tay G.K., Alsafar H. (2018). Multiple genetic variations confer risks for obesity and type 2 diabetes mellitus in arab descendants from UAE. Int. J. Obes..

[20] O’Beirne S.L., Salit J., Rodriguez-Flores J.L., Staudt M.R., Abi Khalil C., Fakhro K.A., Robay A., Ramstetter M.D., Al-Azwani I.K., Malek J.A. (2016). Type 2 diabetes risk allele loci in the Qatari population. PLoS ONE.

[21] Serván P.R. (2013). Obesity and diabetes. Nutr. Hosp..

[22] Vimaleswaran K.S. (2017). Gene–Nutrient Interactions on Metabolic Diseases: Findings from the GeNuIne Collaboration.

[23] Hosseini-Esfahani F., Koochakpoor G., Daneshpour M.S., Sedaghati-Khayat B., Mirmiran P., Azizi F. (2017). Mediterranean dietary pattern adherence modify the association between FTO genetic variations and obesity phenotypes. Nutrients.

[24] Khan S.M., El Hajj Chehadeh S., Abdulrahman M., Osman W., Al Safar H. (2018). Establishing a genetic link between FTO and VDR gene polymorphisms and obesity in the Emirati population. BMC Med. Genet..

[25] Nasreddine L., Akika R., Mailhac A., Tamim H., Zgheib N.K. (2019). The interaction between genetic polymorphisms in FTO and TCF7L2 genes and dietary intake with regard to body mass and composition: An exploratory study. J. Pers. Med..

[26] Aoun C., Hajj A., Hajj F., Papazian T., Khabbaz L.R. (2022). The interaction between genetic polymorphisms in FTO, MC4R and MTHFR genes and adherence to the Mediterranean Diet in relation to obesity. Gene.

[27] Ouhaibi-Djellouli H., Mediene-Benchekor S., Lardjam-Hetraf S.A., Hamani-Medjaoui I., Meroufel D.N., Boulenouar H., Hermant X., Saidi-Mehtar N., Amouyel P., Houti L. (2014). The TCF7L2rs7903146 polymorphism, dietary intakes and type 2 diabetes risk in an Algerian population. BMC Genet..

[28] Platt D.E., Ghassibe-Sabbagh M., Salameh P., Salloum A.K., Haber M., Mouzaya F., Gauguier D., Al-Sarraj Y., El-Shanti H., Zalloua P.A. (2016). Caffeine impact on metabolic syndrome components is modulated by a CYP1A2 variant. Ann. Nutr. Metab..

[29] Campbell M., McKenzie J.E., Sowden A., Katikireddi S.V., Brennan S.E., Ellis S., Hartmann-Boyce J., Ryan R., Shepperd S., Thomas J. (2020). Synthesis without meta-analysis (SWiM) in systematic reviews: Reporting guideline. BMJ.

[30] Downes M.J., Brennan M.L., Williams H.C., Dean R.S. (2016). Development of a critical appraisal tool to assess the quality of cross-sectional studies (AXIS). BMJ Open.

[31] Sterne J.A., Hernán M.A., Reeves B.C., Savović J., Berkman N.D., Viswanathan M., Henry D., Altman D.G., Ansari M.T., Boutron I. (2016). ROBINS-I: A tool for assessing risk of bias in non-randomised studies of interventions. BMJ.

[32] Sterne J.A., Savović J., Page M.J., Elbers R.G., Blencowe N.S., Boutron I., Cates C.J., Cheng H.-Y., Corbett M.S., Eldridge S.M. (2019). RoB 2: A revised tool for assessing risk of bias in randomised trials. BMJ.

[33] Alathari B.E., Bodhini D., Jayashri R., Lakshmipriya N., Shanthi Rani C.S., Sudha V., Lovegrove J.A., Anjana R.M., Mohan V., Radha V. (2020). A Nutrigenetic Approach to Investigate the Relationship between Metabolic Traits and Vitamin D Status in an Asian Indian Population. Nutrients.

[34] Roswall N., Ängquist L., Ahluwalia T.S., Romaguera D., Larsen S.C., Østergaard J.N., Halkjær J., Vimaleswaran K.S., Wareham N.J., Bendinelli B. (2014). Association between Mediterranean and Nordic diet scores and changes in weight and waist circumference: Influence of FTO and TCF7L2 loci. Am. J. Clin. Nutr..

[35] Bodhini D., Gaal S., Shatwan I., Ramya K., Ellahi B., Surendran S., Sudha V., Anjana M.R., Mohan V., Lovegrove J.A. (2017). Interaction between TCF7L2 polymorphism and dietary fat intake on high density lipoprotein cholesterol. PLoS ONE.

[36] Corella D., Coltell O., Sorli J.V., Estruch R., Quiles L., Martínez-González M.Á., Salas-Salvado J., Castañer O., Arós F., Ortega-Calvo M. (2016). Polymorphism of the transcription factor 7-like 2 gene (TCF7L2) interacts with obesity on type-2 diabetes in the PREDIMED study emphasizing the heterogeneity of genetic variants in type-2 diabetes risk prediction: Time for obesity-specific genetic risk scores. Nutrients.

[37] Brouwer I. (2020). The public health rationale for reducing saturated fat intakes: Is a maximum of 10% energy intake a good recommendation?. Nutr. Bull..

[38] Council N.R. (1989). Recommended Dietary Allowances.

[39] Hwalla N., Jomaa L., Hachem F., Kharroubi S., Hamadeh R., Nasreddine L., Naja F. (2021). Promoting sustainable and healthy diets to mitigate food insecurity amidst economic and health crises in lebanon. Front. Nutr..

[40] Fat: The Facts. https://www.nhs.uk/live-well/eat-well/food-types/different-fats-nutrition/#:~:text=Saturated%20fat%20guidelines,of%20saturated%20fat%20a%20day.

[41] Grau K., Cauchi S., Holst C., Astrup A., Martinez J.A., Saris W.H., Blaak E.E., Oppert J.-M., Arner P., Rössner S. (2010). TCF7L2 rs7903146–macronutrient interaction in obese individuals’ responses to a 10-wk randomized hypoenergetic diet. Am. J. Clin. Nutr..

[42] Mattei J., Qi Q., Hu F.B., Sacks F.M., Qi L. (2012). TCF7L2 genetic variants modulate the effect of dietary fat intake on changes in body composition during a weight-loss intervention. Am. J. Clin. Nutr..

[43] Haupt A., Thamer C., Heni M., Ketterer C., Machann J., Schick F., Machicao F., Stefan N., Claussen C.D., Häring H.-U. (2010). Gene variants of TCF7L2 influence weight loss and body composition during lifestyle intervention in a population at risk for type 2 diabetes. Diabetes.

[44] Qi L., Kraft P., Hunter D.J., Hu F.B. (2008). The common obesity variant near MC4R gene is associated with higher intakes of total energy and dietary fat, weight change and diabetes risk in women. Hum. Mol. Genet..

[45] Xi B., Chandak G.R., Shen Y., Wang Q., Zhou D. (2012). Association between common polymorphism near the MC4R gene and obesity risk: A systematic review and meta-analysis. PLoS ONE.

[46] Yu K., Li L., Zhang L., Guo L., Wang C. (2020). Association between MC4R rs17782313 genotype and obesity: A meta-analysis. Gene.

[47] Helou K., El Helou N., Mahfouz M., Mahfouz Y., Salameh P., Harmouche-Karaki M. (2018). Validity and reliability of an adapted arabic version of the long international physical activity questionnaire. BMC Public Health.

[48] Murtagh E., Shalash A., Martin R., Rmeileh N.A. (2021). Measurement and prevalence of adult physical activity levels in Arab countries. Public Health.

[49] Lee P.H., Macfarlane D.J., Lam T.H., Stewart S.M. (2011). Validity of the international physical activity questionnaire short form (IPAQ-SF): A systematic review. Int. J. Behav. Nutr. Phys. Act..

[50] Cherfan M., Blacher J., Asmar R., Chahine M.N., Zeidan R.K., Farah R., Salameh P. (2018). Prevalence and risk factors of hypertension: A nationwide cross-sectional study in Lebanon. J. Clin. Hypertens..

[51] Koochakpoor G., Hosseini-Esfahani F., Daneshpour M.S., Hosseini S.A., Mirmiran P. (2016). Effect of interactions of polymorphisms in the Melanocortin-4 receptor gene with dietary factors on the risk of obesity and Type 2 diabetes: A systematic review. Diabet. Med..

[52] Khodarahmi M., Jafarabadi M.A., Farhangi M.A. (2020). Melanocortin-4 receptor (MC4R) rs17782313 polymorphism interacts with Dietary Approach to Stop Hypertension (DASH) and Mediterranean Dietary Score (MDS) to affect hypothalamic hormones and cardio-metabolic risk factors among obese individuals. Genes Nutr..

[53] Khodarahmi M., Kahroba H., Jafarabadi M.A., Mesgari-Abbasi M., Farhangi M.A. (2020). Dietary quality indices modifies the effects of melanocortin-4 receptor (MC4R) rs17782313 polymorphism on cardio-metabolic risk factors and hypothalamic hormones in obese adults. BMC Cardiovasc. Disord..

[54] Moehlecke M., Canani L.H., Trindade M.R.M., Friedman R., Leitão C.B. (2016). Determinants of body weight regulation in humans. Arch. Endocrinol. Metab..

[55] Yarizadeh H., Bahiraee A., Asadi S., Maddahi N.S., Setayesh L., Casazza K., Mirzaei K. (2020). The interaction between dietary approaches to stop hypertension and MC4R gene variant in predicting cardiovascular risk factors. Int. J. Vitam. Nutr. Res..

[56] Mousavizadeh Z., Hosseini-Esfahani F., Javadi A., Daneshpour M.S., Akbarzadeh M., Javadi M., Mirmrian P., Azizi F. (2020). The interaction between dietary patterns and melanocortin-4 receptor polymorphisms in relation to obesity phenotypes. Obes. Res. Clin. Pract..

[57] Vesnina A., Prosekov A., Kozlova O., Atuchin V. (2020). Genes and eating preferences, their roles in personalized nutrition. Genes.

[58] Barrea L., Annunziata G., Bordoni L., Muscogiuri G., Colao A., Savastano S., Obesity Programs of Nutrition, Education, Research and Assessment (OPERA) Group (2020). Nutrigenetics—Personalized nutrition in obesity and cardiovascular diseases. Int. J. Obes. Suppl..

[59] Wang J., You D., Wang H., Yang Y., Zhang D., Lv J., Luo S., Liao R., Ma L. (2021). Association between homocysteine and obesity: A meta-analysis. J. Evid. Based Med..

[60] Vimaleswaran K.S., Bodhini D., Lakshmipriya N., Ramya K., Anjana R.M., Sudha V., Lovegrove J.A., Kinra S., Mohan V., Radha V. (2016). Interaction between FTO gene variants and lifestyle factors on metabolic traits in an Asian Indian population. Nutr. Metab..

[61] Zhao X., Yang Y., Sun B.-F., Zhao Y.-L., Yang Y.-G. (2014). FTO and obesity: Mechanisms of association. Curr. Diab. Rep..

[62] Tung Y.C.L., Yeo G.S. (2011). From GWAS to biology: Lessons from FTO. Ann. N. Y. Acad. Sci..

[63] Shimaoka I., Kamide K., Ohishi M., Katsuya T., Akasaka H., Saitoh S., Sugimoto K., Oguro R., Congrains A., Fujisawa T. (2010). Association of gene polymorphism of the fat-mass and obesity-associated gene with insulin resistance in Japanese. Hypertens. Res..

[64] Wang H., Dong S., Xu H., Qian J., Yang J. (2012). Genetic variants in FTO associated with metabolic syndrome: A meta-and gene-based analysis. Mol. Biol. Rep..

[65] Bhurosy T., Jeewon R. (2013). Pitfalls of using body mass index (BMI) in assessment of obesity risk. Curr. Res. Nutr. Food Sci. J..

[66] Haddad C., Lahoud N., Akel M., Sacre H., Hajj A., Hallit S., Salameh P. (2020). Knowledge, attitudes, harm perception, and practice related to waterpipe smoking in Lebanon. Environ. Sci. Pollut. Res..

[67] Baalbaki R., Itani L., El Kebbi L., Dehni R., Abbas N., Farsakouri R., Awad D., Tannir H., Kreidieh D., El Masri D. (2019). Association between smoking hookahs (shishas) and higher risk of obesity: A systematic review of population-based studies. J. Cardiovasc. Dev. Dis..

[68] Bonaccio M., Bonanni A.E., Di Castelnuovo A., De Lucia F., Donati M.B., De Gaetano G., Iacoviello L., Investigators M.-s.P. (2012). Low income is associated with poor adherence to a Mediterranean diet and a higher prevalence of obesity: Cross-sectional results from the Moli-sani study. BMJ Open.

[69] Musaiger A.O. (2011). Overweight and obesity in eastern mediterranean region: Prevalence and possible causes. J. Obes..

[70] VanderWeele T.J. (2019). Principles of confounder selection. Eur. J. Epidemiol..

[71] Vimaleswaran K.S. (2021). GeNuIne (gene–nutrient interactions) Collaboration: Towards implementing multi-ethnic population-based nutrigenetic studies of vitamin B12 and D deficiencies and metabolic diseases. Proc. Nutr. Soc..

[72] Leońska-Duniec A., Jastrzębski Z., Zarębska A., Maciejewska A., Ficek K., Cięszczyk P. (2018). Assessing effect of interaction between the FTO A/T polymorphism (rs9939609) and physical activity on obesity-related traits. J. Sport Health Sci..

[73] Isgin-Atici K., Alsulami S., Turan-Demirci B., Surendran S., Sendur S.N., Lay I., Karabulut E., Ellahi B., Lovegrove J.A., Alikasifoglu M. (2021). FTO gene-lifestyle interactions on serum adiponectin concentrations and central obesity in a Turkish population. Int. J. Food Sci. Nutr..

[74] Vimaleswaran K.S., Tachmazidou I., Zhao J.H., Hirschhorn J.N., Dudbridge F., Loos R.J. (2012). Candidate genes for obesity-susceptibility show enriched association within a large genome-wide association study for BMI. Hum. Mol. Genet..

[75] Molag M.L., de Vries J.H., Ocké M.C., Dagnelie P.C., van den Brandt P.A., Jansen M.C., van Staveren W.A., van’t Veer P. (2007). Design characteristics of food frequency questionnaires in relation to their validity. Am. J. Epidemiol..

[76] Cui Q., Xia Y., Wu Q., Chang Q., Niu K., Zhao Y. (2021). A meta-analysis of the reproducibility of food frequency questionnaires in nutritional epidemiological studies. Int. J. Behav. Nutr. Phys. Act..

[77] Gordon D.C. (2016). The Republic of Lebanon: Nation in Jeopardy.

[78] Makhoul N., Wells R., Kaspar H., Shbaklo H., Taher A., Chakar N., Zalloua P. (2005). Genetic heterogeneity of Beta thalassemia in Lebanon reflects historic and recent population migration. Ann. Hum. Genet..

[79] Haddad S. (2009). Lebanon: From consociationalism to conciliation. Natl. Ethn. Politics.

[80] Maktabi R. (1999). The Lebanese census of 1932 revisited. Who are the Lebanese?. Br. J. Middle East. Stud..

[81] Alghamdi J., Padmanabhan S. (2014). Fundamentals of complex trait genetics and association studies. Handb. Pharmacogenomics Stratif. Med..

[82] Kilpeläinen T.O., Qi L., Brage S., Sharp S.J., Sonestedt E., Demerath E., Ahmad T., Mora S., Kaakinen M., Sandholt C.H. (2011). Physical activity attenuates the influence of FTO variants on obesity risk: A meta-analysis of 218,166 adults and 19,268 children. PLoS Med..

[83] Al-Daghri N.M., Al-Attas O.S., Alkharfy K.M., Khan N., Mohammed A.K., Vinodson B., Ansari M.G., Alenad A., Alokail M.S. (2014). Association of VDR-gene variants with factors related to the metabolic syndrome, type 2 diabetes and vitamin D deficiency. Gene.

[84] Cho H.W., Jin H.S., Eom Y.B. (2021). The interaction between FTO rs9939609 and physical activity is associated with a 2-fold reduction in the risk of obesity in Korean population. Am. J. Hum. Biol..

[85] Corella D., Ortega-Azorin C., Sorli J.V., Covas M.I., Carrasco P., Salas-Salvado J., Martínez-González M.Á., Aros F., Lapetra J., Serra-Majem L. (2012). Statistical and biological gene-lifestyle interactions of MC4R and FTO with diet and physical activity on obesity: New effects on alcohol consumption. PLoS ONE.

[86] Vimaleswaran K.S., Ängquist L., Hansen R.D., Van Der A D.L., Bouatia-Naji N., Holst C., Tjønneland A., Overvad K., Jakobsen M.U., Boeing H. (2012). Association between FTO variant and change in body weight and its interaction with dietary factors: The DiOGenes study. Obesity.

[87] Sonestedt E., Roos C., Gullberg B., Ericson U., Wirfält E., Orho-Melander M. (2009). Fat and carbohydrate intake modify the association between genetic variation in the FTO genotype and obesity. Am. J. Clin. Nutr..

[88] Petkeviciene J., Smalinskiene A., Klumbiene J., Petkevicius V., Kriaucioniene V., Lesauskaite V. (2016). Physical activity, but not dietary intake, attenuates the effect of the FTO rs9939609 polymorphism on obesity and metabolic syndrome in Lithuanian adult population. Public Health.

[89] Vimaleswaran K., Power C., Hyppönen E. (2014). Interaction between vitamin D receptor gene polymorphisms and 25-hydroxyvitamin D concentrations on metabolic and cardiovascular disease outcomes. Diabetes Metab..

[90] Uitterlinden A.G., Fang Y., Van Meurs J.B., Pols H.A., Van Leeuwen J.P. (2004). Genetics and biology of vitamin D receptor polymorphisms. Gene.

[91] Alathari B.E., Sabta A.A., Kalpana C.A., Vimaleswaran K.S. (2020). Vitamin D pathway-related gene polymorphisms and their association with metabolic diseases: A literature review. J. Diabetes Metab. Disord..

[92] Hasan H.A., AbuOdeh R.O., Muda W., Mohamed H., Samsudin A.R. (2017). Association of Vitamin D receptor gene polymorphisms with metabolic syndrome and its components among adult Arabs from the United Arab Emirates. Diabetes Metab. Syndr..

[93] Hemkens L.G., Ewald H., Naudet F., Ladanie A., Shaw J.G., Sajeev G., Ioannidis J.P. (2018). Interpretation of epidemiologic studies very often lacked adequate consideration of confounding. J. Clin. Epidemiol..

[94] Van Dyke N., Drinkwater E.J., Rachele J.N. (2022). Improving the accuracy of self-reported height and weight in surveys: An experimental study. BMC Med. Res. Methodol..

[95] Naska A., Lagiou A., Lagiou P. (2017). Dietary assessment methods in epidemiological research: Current state of the art and future prospects. F1000Research.

[96] Tucker K.L. (2007). Assessment of usual dietary intake in population studies of gene–diet interaction. Nutr. Metab. Cardiovasc. Dis..

[97] Culliford A.E., Bradbury J., Medici E.B. (2023). Improving Communication of the UK Sustainable Healthy Dietary Guidelines the Eatwell Guide: A Rapid Review. Sustainability.

[98] Vimaleswaran K.S. (2020). A nutrigenetics approach to study the impact of genetic and lifestyle factors on cardiometabolic traits in various ethnic groups: Findings from the GeNuIne Collaboration. Proc. Nutr. Soc..

[99] Amin N., Byrne E., Johnson J., Chenevix-Trench G., Walter S., Nolte I., Vink J., Rawal R., Mangino M., Teumer A. (2012). Genome-wide association analysis of coffee drinking suggests association with CYP1A1/CYP1A2 and NRCAM. Mol. Psychiatry.

[100] Tornio A., Backman J.T. (2018). Cytochrome P450 in pharmacogenetics: An update. Adv. Pharmacol..

[101] Liu J., Sridhar J., Foroozesh M. (2013). Cytochrome P450 family 1 inhibitors and structure-activity relationships. Molecules.

[102] Nebert D.W., Dalton T.P. (2006). The role of cytochrome P450 enzymes in endogenous signalling pathways and environmental carcinogenesis. Nat. Rev. Cancer.

[103] Wang C. (2020). Revealing obesity through diet-gene interactions. Health Sci. Inq..

[104] Palatini P., Benetti E., Mos L., Garavelli G., Mazzer A., Cozzio S., Fania C., Casiglia E. (2015). Association of coffee consumption and CYP1A2 polymorphism with risk of impaired fasting glucose in hypertensive patients. Eur. J. Epidemiol..

[105] Robertson T.M., Clifford M.N., Penson S., Williams P., Robertson M.D. (2018). Postprandial glycaemic and lipaemic responses to chronic coffee consumption may be modulated by CYP1A2 polymorphisms. Br. J. Nutr..

[106] Banks N., Tomko P., Colquhoun R., Muddle T., Emerson S., Jenkins N. (2019). Genetic polymorphisms in ADORA2A and CYP1A2 influence caffeine’s effect on postprandial glycaemia. Sci. Rep..

[107] Hüls A., Krämer U., Carlsten C., Schikowski T., Ickstadt K., Schwender H. (2017). Comparison of weighting approaches for genetic risk scores in gene-environment interaction studies. BMC Genet..

