# Supplementary material for: A Systematic Review of the Gene–Lifestyle Interactions on Metabolic Disease-Related Outcomes in Arab Populations
Source: Nutrients. 2024 Aug 1;16(15):2519. doi: 10.3390/nu16152519 (PMC11314532; doi:10.3390/nu16152519)
Supplement: Supplementary file 1 [file nutrients-16-02519-s001.zip › nutrients-3081744-supplementary.pdf]

Table S1: Search Strings

Search engine	Search string	Number of hits
PubMed	(nutrigenetic) AND (Arab)	10
	((nutrigenetic) AND (middle eastern))	7
	(gene-diet interaction) AND (middle eastern)	2
	((polymorphism) AND (dietary patterns)) AND (Arab)	2
	((polymorphism) AND (dietary intake)) AND (Arab)	5
	((gene) AND (dietary intake)) AND (Arab)	22
	((genetic variant) AND (dietary intake)) AND (Arab)	7
	((gene polymorphism) AND (obesity)) AND (Arab)	59
	((genetic variant) AND (physical activity)) AND (Arab)	12
	(((gene variant) AND (lifestyle) AND (Interaction))	702
	((((polymorphism* OR gene OR SNP OR single nucleotide polymorphism OR genetic variation OR genetic variant OR GRS OR genetic risk score OR PRS OR polygenic risk score) AND ("gene-diet interaction" OR "diet-gene interaction" OR "gene- diet" OR "diet-gene" OR "gene and diet" OR "diet and gene" OR "SNP-diet interaction" OR "diet- SNP interaction" OR "SNP-diet" OR "diet-SNP" OR "SNP and diet" OR "diet and SNP" OR "gene- nutrient interaction" OR "nutrient-gene interaction" OR "gene-nutrient" OR "nutrient-gene" OR "gene and nutrient" OR "nutrient and gene" OR "gene-lifestyle interaction" OR "lifestyle-gene interaction" OR "gene-lifestyle" OR "lifestyle-gene" OR "gene and lifestyle" OR "lifestyle and gene" OR "gene-environment interaction" OR "environment-gene interaction" OR "gene-environment" OR "environment-gene" OR "gene and environment" OR "environment and gene")) AND (carbohydrate OR protein OR fat OR fiber OR fiber OR sugar OR SFA OR saturated fat OR monounsaturated fat OR polyunsaturated fat OR MUFA OR PUFA OR diet OR B12 OR vitamin D OR amino acid OR polyphenol OR egg intake OR coffee OR caffeine intake OR green tea OR alcohol intake OR meat intake OR energy intake OR food)) AND (Obesity OR weight OR BMI OR waist circumference OR waist hip ratio OR hip circumference OR adiposity OR metabolic diseases OR lifestyle diseases OR diabetes OR T2DM OR body fat OR body composition)) AND (Algeria OR Bahrain OR Comoros OR Djibouti OR Egypt OR Iraq OR Jordon OR Kuwait OR Lebanon OR Libya OR Mauritania OR Morocco OR Oman OR Palestinian OR Qatar OR Saudi Arabia OR Somalia OR	1483

Risk of Bias Assessment:

Appraisal tool for Cross-sectional Studies (AXIS)

Introduction

1. Were the aims/ objectives of the study clear?

Methods

2. Was the study design appropriate for the stated aim(s)?
3. Was the sample size justified?
4. Was the target/reference population clearly defined? (Is it clear who the research was about?)
5. Was the sample frame taken from an appropriate population base so that it closely represented the target/reference population under investigation?
6. Was the selection process likely to select subjects/participants that were representative of the target/reference population under investigation?
7. Were measures undertaken to address and categorize non-responders?
8. Were the risk factor and outcome variables measured appropriate to the aims of the study?
9. Were the risk factor and outcome variables measured correctly using instruments/ measurements that had been trialed, piloted, or published previously? (Only dietary, nutritional, lifestyle, and physical activity assessment were evaluated.)
10. Is it clear what was used to determined statistical significance and/or precision estimates? (e.g., p-values, CIs)
11. Were the methods (including statistical methods) sufficiently described to enable them to be repeated?

Results

12. Were the basic data adequately described?
13. Does the response rate raise concerns about non-response bias?
14. If appropriate, was information about non-responders described?
15. Were the results internally consistent?
16. Were the results for the analyses described in the methods presented?

Discussion

17. Was the author´s discussion and conclusions justified by the results?
18. Were the limitations of the study discussed?
19. Were there any funding sources or conflicts that may affect the authors' interpretations of the results?
20. Was ethical approval or consent of participants attained?

Table S2: Summary Outcome of Assessment with the AXIS.

	Nasreddine et al. 2019	Aoun et al. 2022	Platt et al. 2016	Ouhaibi-Djellouli et al. 2014
	Introduction			
1)	Y	Y	Y	Y
	Methods			
2)	Y	Y	Y	Y
3)	Y	N	N	Y
4)	Y	Y	Y	Y
5)	N	N	N	Y
6)	Y	Y	Y	Y
7)	Y	Y	Y	Y
8)	Y	Y	N	Y
9)	Y	Y	Y	Y
10)	Y	Y	Y	Y
11)	Y	Y	N	Y
	Results			
12)	Y	Y	Y	Y
13)	N	N	N	N
14)	Y	Y	Y	Y
15)	Y	Y	Y	Y
16)	Y	Y	Y	Y
	Discussion			
17)	Y	Y	Y	Y
18)	Y	Y	N	Y
19)	N	N	N	N
20)	Y	Y	Y	Y

Note: numbered questions are listed. Y = Yes, N = No.

Table S3: Assessment with the AXIS

	Intro	Method										Results					Discussion			
	1	2	3	4	5	6	7	8	9	10	11	12	13	14	15	16	17	18	19	20.
Nasreddine et al. 2019	Y	Y	Y	Y	N	Y	Y	Y	Y	Y	Y	Y	N	Y	Y	Y	Y	Y	N	Y
Comment					Sample was not representative of the population															
Aoun et al. 2022	Y	Y	N	Y	N	Y	Y	Y	Y	Y	Y	Y	N	Y	Y	Y	Y	Y	N	Y
Comment					Sample was not representative of the population															
Platt et al. 2016	Y	Y	N	Y	N	Y	Y	N	N	Y	N	Y	N	Y	Y	Y	Y	N	N	Y
Comment					Sample was not			The measures of coffee were asked	No1larifycation was provided regarding									Did not discuss		

					representative of the population			as categories. This variable could have been treated as a continuous variable, thus resulting in a loss of statistical power.	development, content, administration method (self-administered or as part of an interview), or whether trained personnel conducted the questionnaire.									internal limitations.		
Ouhaibi-Djellouli et al. 2014	Y	Y	Y	Y	Y	Y	Y	Y	Y	Y	Y	Y	N	Y	Y	Y	Y	Y	N	Y
Comment																				

Note: Questions are listed above; Table S2 summarizes Table S3. Y = Yes, N = No.

Table S4. Assessment using the Risk of Bias in Non-Randomized Studies of Interventions (ROBINS-I)

Study: Khan et al. 2018	Answer	[Description]
Bias due to confounding		
1.1 Is there potential for confounding of the effect of exposure in this study? If N or PN to 1.1: the study can be considered to be at low risk of bias due to confounding, and no further signaling questions need be considered Y / PY / PN / N	PY	A lifestyle questionnaire in which physically active was asked as a dichotomic question (yes/no) with no further clarification, with adjustment only for age and systolic blood pressure, thus not considering rest of confounders in the modeling.
If Y/PY to 1.1, answer 2.1 and 1.3 to determine whether there is a need to assess time-varying confounding:		
1.2. If Y or PY to 1.1: Was the analysis based on splitting or followup time according to exposure received?	N	
If N or PN to 1.2, answer questions 1.4 to 1.6, which relate to baseline confounding		
1.3. If Y or PY to 1.2: Were exposure discontinuations or switches likely to be related to factors that are prognostic for the outcome?	Y	The extent of obesity may be involved in a discontinuation or switches on being physically active.
1.4. Did the authors use an appropriate analysis method that adjusted for all the critically important confounding areas?	N	The reported interaction was only adjusted for age and systolic blood pressure, but not sex and T2D. This was not explained in the methodology.
1.6. Did the authors avoid adjusting for post-exposure variables?	PY	They did not adjust for relevant variables such as sex.
If Y or PY to 1.3, answer questions 1.7 and 1.8, which relate to time-varying confounding		
1.7. Did the authors use an appropriate analysis method that adjusted for all the critically important confounding areas and for time-varying confounding?	N	They did not adjust for relevant variables such as sex.
1.8. If Y or PY to 1.7: Were confounding areas that were adjusted for measured validly and reliably by the variables available in this study?	PY	
Bias in selection of participants into the study		
2.1. Was selection of participants in the study (or in the analysis) based on variables measured after the start of the exposure?	N	
If N or PN to 2.1 go to 2.4		

2.4 Do start of followup and start of exposure coincide for most participants?	Y	
Bias in classification of exposures		
3.1 Is exposure status well defined?	N	physically active was asked as a dichotomic question (yes/no)
3.2 Did entry into the study begin with start of the exposure?	N	
3.3 Was information used to define exposure status recorded prior to outcome assessment?		
3.4 Could classification of exposure status have been affected by knowledge of the outcome or risk of the outcome?		
3.5 Were exposure assessment methods robust (including methods used to input data)?		
Bias due to departures from intended exposures		
4.1. Is there concern that changes in exposure status occurred among participants?	N	
Bias due to departures from intended exposures		
5.1 Were there missing outcome data?	N	
5.2 Were participants excluded due to missing data on exposure status?	N	
5.3 Were participants excluded due to missing data on other variables needed for the analysis?	N	
Bias in measurement of outcomes		
6.1 Could the outcome measure have been influenced by knowledge of the exposure received?	N	
6.2 Was the outcome measure sensitive?	N	
6.3 Were outcome assessors unaware of the exposure received by study participants?	PN	
6.4 Were the methods of outcome assessment comparable across exposure groups?	Y	
6.5 Were any systematic errors in measurement of the outcome unrelated to exposure received?	N	

Y: Yes, PY: Probably Yes, PN: Probably No, N: No.
